# A Systematic Review Exploring the Social Cognitive Theory of Self-Regulation as a Framework for Chronic Health Condition Interventions

**DOI:** 10.1371/journal.pone.0134977

**Published:** 2015-08-07

**Authors:** Michelle E. Tougas, Jill A. Hayden, Patrick J. McGrath, Anna Huguet, Sharlene Rozario

**Affiliations:** 1 Community Health and Epidemiology, Dalhousie University, Halifax, Nova Scotia, Canada; 2 Psychology and Neuroscience, Dalhousie University, Halifax, Nova Scotia, Canada; 3 Centre for Research in Family Health, IWK Health Centre, Halifax, Nova Scotia, Canada; 4 Canada Research Chair, Dalhousie University, Halifax, Nova Scotia, Canada; 5 Science, Pediatrics, and Psychiatry, Dalhousie University, Halifax, Nova Scotia, Canada; 6 Capital District Health Authority, Halifax, Nova Scotia, Canada; University of Geneva, SWITZERLAND

## Abstract

**Background:**

Theory is often recommended as a framework for guiding hypothesized mechanisms of treatment effect. However, there is limited guidance about how to use theory in intervention development.

**Methods:**

We conducted a systematic review to provide an exemplar review evaluating the extent to which use of theory is identified and incorporated within existing interventions. We searched electronic databases PubMed, PsycINFO, CENTRAL, and EMBASE from inception to May 2014. We searched clinicaltrials.gov for registered protocols, reference lists of relevant systematic reviews and included studies, and conducted a citation search in Web of Science. We included peer-reviewed publications of interventions that referenced the social cognitive theory of self-regulation as a framework for interventions to manage chronic health conditions. Two reviewers independently assessed articles for eligibility. We contacted all authors of included studies for information detailing intervention content. We describe how often theory mechanisms were addressed by interventions, and report intervention characteristics used to address theory.

**Results:**

Of 202 articles that reported using the social cognitive theory of self-regulation, 52% failed to incorporate self-monitoring, a main theory component, and were therefore excluded. We included 35 interventions that adequately used the theory framework. Intervention characteristics were often poorly reported in peer-reviewed publications, 21 of 35 interventions incorporated characteristics that addressed each of the main theory components. Each intervention addressed, on average, six of eight self-monitoring mechanisms, two of five self-judgement mechanisms, and one of three self-evaluation mechanisms. The self-monitoring mechanisms ‘Feedback’ and ‘Consistency’ were addressed by all interventions, whereas the self-evaluation mechanisms ‘Self-incentives’ and ‘External rewards’ were addressed by six and four interventions, respectively. The present review establishes that systematic review is a feasible method of identifying use of theory as a conceptual framework for existing interventions. We identified the social cognitive theory of self-regulation as a feasible framework to guide intervention development for chronic health conditions.

## Introduction

Theory can provide a framework for guiding the development and implementation of health interventions. The use of theory is recommended by the UK Medical Research Council to provide hypotheses of specific mechanisms and interactions [1–4] during the first phase in the development of interventions [5]. Theory may be particularly useful for interventions that encompass several interacting active management strategies, and are often difficult to evaluate and reproduce, for example interventions directed at chronic health conditions [6]. Current recommendations to use theory early in the design of interventions, however, do not specifically describe how to incorporate theory in the development process. In health behaviour literature, systematic reviews report that only 22–36% of interventions describe using any theoretical framework or theory components to guide their development [7,8].

The importance of managing chronic health conditions is evident by their increasing prevalence and leading role in worldwide morbidity and mortality [9]. Many of these conditions can be prevented, treated, and managed through behaviour change interventions, which provide individuals with the skills to have control over and improve their health [7,9]. Using theory to develop chronic health interventions can help to identify what behaviour change mechanisms are influential for improving health outcomes.

The social cognitive theory proposed by Bandura (1986) [10], is one of the most common behaviour change theories applied in the management of chronic health conditions [7]. One concept of the theory focuses on the importance of self-regulation as a source of behaviour change, which is broken down into three core components: self-monitoring, self-judgement, and self-evaluation [10,11]. Evidence from randomized controlled trials based on the social cognitive theory of self-regulation supports the clinical benefits of interventions based on this theory for health outcomes in asthma [12], arthritis [13], weight loss [14], and cardiac rehabilitation [15]. These findings suggest that interventions based on the social cognitive theory of self-regulation can be useful for improving outcomes in some chronic health conditions. Nonetheless, the selection of the specific theory components and associated mechanisms that have been chosen to be addressed with particular intervention characteristics remains unclear.

The objectives of this systematic review of the literature were to evaluate the extent to which theory has been used in the development of existing interventions, and to provide an example of how literature can be systematically reviewed to explore use of theory as a framework for existing interventions. We explored how researchers use the social cognitive theory of self-regulation to inform the management of chronic health conditions. We assessed peer-reviewed publications that reported the evaluation of interventions to identify which theory components and mechanisms were implemented most often, and how the interventions addressed each of the theory mechanisms.

## Materials and Methods

### Literature Search and Data Sources

A protocol is available upon request to the first author. The Preferred Reporting Items for Systematic Reviews and Meta-analysis (PRISMA) [16] was followed for reporting the systematic review (S1 Table). We used multiple search strategies to identify relevant studies. First we searched electronic databases PubMed, PsycINFO, and EMBASE from inception to May 2014, using a search strategy of MeSH terms, keywords reflecting the health conditions of interest, and terms associated with the social cognitive theory of self-regulation (see S1 Text for full PubMed search strategy), we searched the Cochrane Central Register of Controlled Trials for completed controlled trials, and the trial registry clinicaltrials.gov for relevant protocols, which were followed up for published studies. Second, we conducted a citation search in Web of Science to identify studies citing Bandura’s first report of the social cognitive theory of self-regulation [10], or Bandura’s first paper [11] that comprehensively described the theory components and mechanisms. Third, we examined the reference lists of all included studies, and the reference lists of studies included in systematic reviews of self-monitoring interventions identified through a scoping literature search [17–20]. We searched PubMed for available published studies of identified protocols that met our inclusion criteria by searching for studies published by the protocols’ first author, and searching for publications using the intervention’s name, when available. All of the retrieved citations were imported into an EndNote database, where duplicate citations across data sources were identified and removed.

### Inclusion and Exclusion Criteria

We included peer-reviewed publications of studies reporting interventions for chronic health conditions based on the social cognitive theory of self-regulation meeting all of the inclusion criteria described below.

For a homogenous population, we selected studies including participants with chronic health conditions with similar characteristics, which were non-communicable, long-lasting, with a constant non- or slowly-progressive course, and with associated health episodes or behaviour suitable for monitoring. We therefore included the chronic health conditions arthritis, asthma, chronic pain, diabetes, heart disease, and overweight/obesity.

We included studies reporting interventions that stated being designed using the social cognitive theory of self-regulation as the theoretical basis for the intervention, used self-monitoring as an intervention characteristic, and cited one of the main theory publications [10,11]. The social cognitive theory of self-regulation proposes that three main components of the theory, self-monitoring, self-judgement, and self-evaluation, contribute to self-regulation, and influence successful behaviour change. The theory suggests that specific mechanisms related to each of these three main components may be directly associated with successful self-monitoring, self-judgement, and self-evaluation, and influence subsequent behaviour change. The theory identifies self-monitoring as the first and most important step to initiating and informing appropriate self-regulation and behaviour change. We included in this systematic review only interventions that explicitly recommended and expected participants to self-monitor by observing, tracking, and/or recording their own behaviour as a core component of the interventions.

We included peer-reviewed publications of studies that reported the evaluation of relevant interventions, including evaluation of the usability, feasibility, or efficacy/effectiveness of the interventions using observational or experimental designs.

We excluded studies that: 1) cited the social cognitive theory of self-regulation but did not report evaluation of an intervention, 2) were available as conference proceedings, abstracts, case studies, theses, reviews, summaries, commentaries, editorials, letters to the editor, or study protocols without published data, 3) used proxies of the population of interest (e.g., parental administration of an intervention designed to change child behaviour), or used non-human subjects, 4) were not published in English.

### Selection Process

We used two screening phases to identify studies reporting potentially relevant interventions from titles and abstracts. First, one reviewer (MT) conducted an initial title and abstract screen to eliminate readily identifiable ineligible types of publications, and studies conducted in clearly irrelevant health conditions. Second, two reviewers (MT, SR) independently screened the remaining titles and abstracts to determine study design eligibility for full text review.

We also used two screening phases at the full text level. In the first phase one reviewer (MT) screened the full-text articles to identify studies citing the social cognitive theory of self-regulation [10,11]. In the second phase, both reviewers independently applied selection criteria to the remaining full text articles. We report the final number of studies (and independent interventions) identified; duplicate publications (i.e., studies reporting the same intervention) were reviewed for any additional information and used to complete data extraction. We calculated interrater reliability for the second phase of title/abstract and full text screening levels using Cohen’s Kappa [21], and considered Kappa between 0.41–0.60 an indication of moderate level of agreement [22]. Discrepancies were discussed and resolved, using consultation with a third reviewer (JH) when necessary.

### Data Extraction

To supplement information extracted about the content of included interventions, we searched for related publications, protocols, guidelines, and web-based resources. We contacted first authors of included interventions for access to either an intervention manual describing the intervention content, or an intervention guide/outline if a manual was not available. When multiple study publications were identified for one intervention, we contacted the first author of the earliest publication retrieved. When our searches identified information from multiple study publications about the same intervention, we combined this information during extraction. We considered interventions that were published by the same research team across multiple study publications distinct from one another only when at least one main theory component within the intervention was added or removed.

We extracted two types of data from included interventions: 1) study characteristics, and 2) theory-related intervention characteristics.

One reviewer (MT) extracted data on study characteristics, including: study authorship, health condition, inclusion/exclusion criteria, age group, study design, intervention objectives, intervention duration, intervention delivery format, general intervention content, and any additional theories guiding intervention development. A second reviewer (SR) checked the extracted data for accuracy.

We extracted data on the intervention characteristics related to the social cognitive theory of self-regulation. We initially followed a consensus procedure to define the extraction process. We created an outline based on Bandura’s two publications that describe the three main components of the theory that are related to successful self-regulation and behaviour change: self-monitoring, self-judgement, and self-evaluation [10,11]. Self-monitoring involves attention to, noticing, and tracking personal behaviour, which may inform self-judgement. Self-judgement is the process of applying personal standards and values to judge monitored behaviour. Finally, the theory proposes that self-evaluation of monitored behaviour may occur as a result of judgement and directly inform subsequent action, leading all three components to contribute to self-regulation and behaviour change. Within each of the three components, the theory proposes specific mechanisms that may directly influence self-monitoring, self-judgement and self-evaluation, Table 1.

**Table 1:** Description of the self-regulatory mechanism proposed by the social cognitive theory of self-regulation [10,11].

Component	Mechanism	Descriptions
**Self-monitoring**	**Feedback**	Providing evidence of behaviour change progress
	**Temporal proximity**	Monitoring behaviour close in time to when it occurs
	**Consistency**	Self-monitoring regularly rather than intermittently
	**Focus on success**	Attending to achievement rather than failure
	**Value of behaviour**	Self-monitoring behaviour with perceived importance
	**Control**	Self-monitoring behaviour easy to deliberately modify
	**Motivation**	Desiring to change the monitored behaviour
	**Self-diagnosis**	Gaining insight through identifying behaviour patterns
**Self-judgement**	**Social comparison**	Relating self-progress with peers in similar situations
	**Self-comparison**	Contrasting ongoing progress with previous behaviour
	**Statistical comparison**	Evaluating progress by contrasting with normative data
	**Modeling**	Examples of others successful in changing behaviour
	**Education/ reaction**	Other’s opinions or responses to inform judgement
**Self-evaluation**	**Self-satisfaction**	Gaining self-respect for goal completion or progress
	**Self-incentives**	Setting personal rewards for achieving progress
	**External rewards**	Setting tangible benefits for completion of a task or goal

We used the theory definitions to identify intervention characteristics that addressed each of the specific mechanisms proposed to be associated with the three theory components. Three reviewers (MT, JH, AH) independently reviewed four selected interventions that comprehensively described included intervention characteristics. The reviewers used the mechanism definitions to independently code the intervention characteristics that addressed each theory mechanism. We explored agreements and disagreements across the reviewers and reached consensus through discussion about the types of intervention characteristics that were applicable to each of the theory mechanisms. We revised the extraction guide with descriptions to specify the type of intervention characteristics that addressed each theory mechanism (S2 Table). We used the extraction guide to develop the final extraction form. Subsequently, two reviewers (MT, SR) extracted the intervention characteristics from remaining studies using the extraction form.

### Risk of Bias Assessment

Two reviewers (MT, SR) independently assessed the risk of bias of the extracted studies. We assessed randomized controlled trials for internal validity, using the Cochrane Collaboration’s Risk of Bias tool to assess the selection bias, performance bias, detection bias, attrition bias, and reporting bias (S3 Table) [23]. Following recommendations by Higgins [24], we judged studies to have an overall ‘high risk of bias’ when at least one of the key domains had a high risk of bias, ‘unclear’ risk of bias when any of the key domains were rated as unclear risk, and ‘low’ risk of bias when all domains were rated as low risk [24]. We used the Quality in Prognosis Studies (QUIPS) tool [25] to assess risk of bias for observational studies, while considering the following domains: participant selection, attrition, outcome measurement, confounding, and analysis/reporting (see reference for the full published tool). We rated each domain as high, moderate, or low risk of bias, and judged the overall internal validity across domains by judging studies as low risk only when all of the domains were rated as low, and as high risk of bias when any of the domains were rated as moderate or high. We calculated interrater reliability for risk of bias assessment using Cohen’s Kappa [21] and considered Kappa between 0.41–0.60 an indication of a moderate level of agreement [22]. A third reviewer was available for consultation about any unresolved discrepancies, however, consultation was not necessary.

### Data Synthesis

We report frequency of use of the social cognitive theory of self-regulation in the development of each intervention, including how often: 1) interventions addressed all three theory components together, two theory components, or only self-monitoring, 2) interventions had characteristics belonging to each specific theory component, and 3) interventions included characteristics belonging to each specific theory mechanism within each of the three theory components. We considered interventions to address a specific mechanism when at least one intervention characteristic included the theory element proposed to be associated with that mechanism, Table 1. We rated interventions as addressing a specific theory component when at least one mechanism related to that component was judged to be present. Interventions addressed all three theory components when at least one intervention characteristic was judged to be present for at least one mechanism related to each of the self-monitoring, self-judgement, and self-evaluation theory components. Interventions addressed only two theory components when at least one intervention characteristic related to at least one mechanism was present for self-monitoring, along with one other theory component (either self-judgement or self-evaluation). To illustrate the types of intervention characteristics that we judged as addressing the theory mechanisms, we provide some examples of characteristics from the included interventions that clearly represented the descriptions in our extraction guide. We explored whether the study risk of bias impacted how often the main theory components were addressed across the interventions. We present subgroup information about how many theory components were addressed by interventions for each assessed risk of bias.

## Results

### Description of Included Interventions

**Fig 1:**
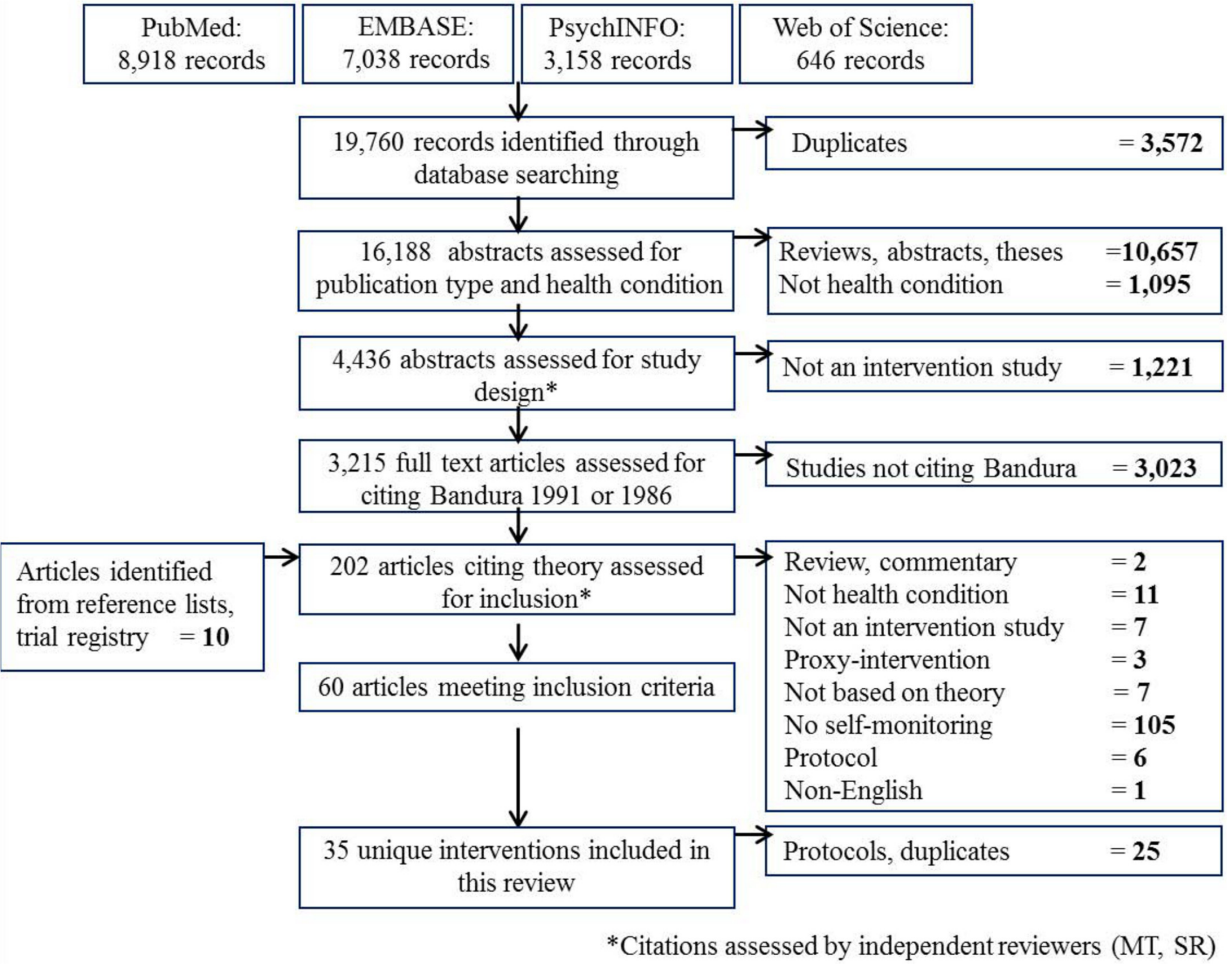
Flow diagram. Flow diagram of title/abstract and full-text screening process to identify interventions included in the review.

We identified and screened 16,188 independent titles and abstracts (Fig 1). We excluded the majority of citations because of ineligible study design. We assessed full text publications for 202 potentially relevant studies that reported interventions for the health conditions of interest, and cited the social cognitive theory of self-regulation [10,11]. Of studies reporting interventions citing the theory, 105 (52%) were excluded because they did not include self-monitoring as a core component of the intervention and therefore did not appropriately address the self-regulation concept of the theory. Our interrater reliability for study selection was moderate for title and abstract, as well as for full-text screening, with a Kappa of 0.60 and 0.55, respectively.

We identified 60 relevant studies, which reported 35 unique interventions developed using the social cognitive theory of self-regulation as a conceptual framework, Table 2. Overweight/obesity was the most common type of health condition addressed by the interventions (14/35) (Table 3, S4 Table). Interventions lasted from four weeks to twelve months, and were delivered through individual-based (17 interventions), group-based (16 interventions), or mixed settings (2 interventions). Most interventions were evaluated using a randomized controlled trial study design (33/35), with an equal distribution of studies assessed as low (13 interventions), high (11 interventions), and unclear (11 interventions) risk of bias. Our interrater reliability for risk of bias assessment was moderate with a Kappa of 0.59. Of the 34 intervention authors contacted (regarding 35 interventions), 11 provided additional information: five provided access to an intervention manual, two provided intervention outlines, and four referred to previous publications.

**Table 2:** Summary of intervention characteristics included in the review.

First author	Population^a^	Duration	Delivery	Instructor condition
**Overweight/obesity**				
Annesi [26]	Severely obese	26 weeks	Group-based	Wellness specialist
Burke [27]	Overweight	18 months	Group-based	Physiotherapist
Collins [28]	Overweight	12 weeks	Web-based	None
Gallagher [29]	Overweight	16 weeks	Group-based	Multidisciplinary
Gray [14]	Overweight men	12 weeks	Group-based	Community coach
Hollis [30]	Overweight	24 weeks	Group-based	Nutritionist, counselor
Kiernan [31]	Overweight women	20 weeks	Group-based	Intervention staff
Ma [32]	Overweight	12 weeks	Group-based	Dietitian, fitness coach
Mockus [33]	Overweight children	20 weeks	Face-to-face	Counselor
Morgan [34]	Overweight men	12 weeks	Web-based	None
Morgan [35]	Overweight fathers	12 weeks	Group-based	Study investigator
Patrick [36]	Overweight	12 months	Web-based	None
Short [37]	Overweight men	9 months	Web-based	None
Shuger [38]	Overweight	14 weeks	Group-based	Intervention staff
**Diabetes**				
Lawler [39]	Type II diabetes	18 months	Telephone	Counselor
Liebreich [40]	Type II diabetes	12 weeks	Web-based	None
Miller [41]	Type II diabetes	10 weeks	Group-based	Dietitian
Nansel [42]	Type I diabetic youth	8 weeks	Face-to-face	Intervention staff
Tan [43]	Type I or II diabetes	12 weeks	Face-to-face	Study investigator
Tudor-Locke [44]	Type II diabetes	16 weeks	Group-based	Physical activity experts
Van Dyck [45]	Type II diabetes	24 weeks	Telephone	Psychologist
**Heart disease**				
Furber [15]	Cardiac patients	6 weeks	Telephone	Not reported
Moore [46]	Recent cardiac event	12 weeks	Group-based	Nurse
Padula [47]	Heart failure	12 weeks	Face-to-face	Nurse
Peterson [48]	Coronary artery disease	12 months	Telephone	Intervention staff
Pinto [49]	Cardiac rehabilitation	14 weeks	Telephone	Intervention staff
Shao [50]	Heart failure	12 weeks	Face-to-face, phone	Not reported
**Arthritis**				
Hughes [51]	Low body osteoarthritis	8 weeks	Group-based	Physical therapists
Kovar [13]	Knee osteoarthritis	8 weeks	Group-based	Intervention staff
Manning [52]	Upper body arthritis	12 weeks	Group-based	Physiotherapist
Shigaki [53]	Rheumatoid arthritis	10 weeks	Web-based	None
**Asthma**				
Baptist [12]	Asthma	6 weeks	Group, phone	Health educator
Burkhart [54]	Asthma, children	16 weeks	Face-to-face	Nurse
Clark [55]	Asthma, women	24 weeks	Telephone	Nurse
McGhan [56]	Asthma, children	6 weeks	Group-based	Nursing students

^a^Adult populations unless otherwise stated

**Table 3:** Summary of design and characteristics of included interventions (n = 35).

Intervention design and study characteristics		Number of interventions (%^a^)
**Health condition**	Overweight/obesity	14 (40%)
	Diabetes	7 (20%)
	Heart disease	6 (17%)
	Arthritis	4 (11%)
	Asthma	4 (11%)
	Pain	0 (0%)
**Study age group**	Adults	31 (89%)
	Children or adolescents	4 (11%)
**Method(s) of intervention delivery**	Group-based	16 (46%)
	Individual telephone contact	6 (17%)
	Internet-based	6 (17%)
	Individual face-to-face	5 (14%)
	Individual face-to-face + telephone contact	1 (3%)
	Group-based + telephone contact	1 (3%)
**Study design**	Experimental (randomized controlled trials)	33 (94%)
	Observational	2 (6%)
**Study overall risk of bias rating**	Low	13 (37%)
	High	11 (31%)
	Unclear	11 (31%)
**Use of theory**	All three theory components	21 (60%)
	Two theory components	14 (40%)
	Only self-monitoring theory component	0 (0%)

^a^All percentages are rounded to the nearest whole number

### Overview of Theory Component Use

Twenty-one of thirty-five interventions incorporated all three of the main theory components by including at least one intervention characteristic that addressed one or more mechanism for self-monitoring, self-judgement, and self-evaluation. Based on information available in peer-reviewed study publications, only 17 interventions were initially identified that used characteristics addressing mechanisms related to self-evaluation. Additional information from four of five intervention manuals provided by authors, were found to incorporate self-evaluation characteristics that were not previously identified, resulting in 21 interventions identified to address all three of the main theory components.

#### Self-monitoring

Each intervention addressed an average of 6.2 of the 8 self-monitoring mechanisms. All mechanisms were frequently used across interventions, with an average of 24 interventions addressing each of the 8 self-monitoring mechanisms. The mechanism ‘Self-diagnosis’ was incorporated least often, by 14 interventions, followed by ‘Focus on success’ and ‘Temporal proximity’, each addressed by 16 interventions. Table 4 describes the intervention characteristics that addressed each self-monitoring theory mechanism. S5 Table provides a summary of how many self-monitoring mechanisms were addressed by each of the individual interventions.

**Table 4:** Characteristics of interventions included in the review (n = 35) addressing self-monitoring mechanisms as proposed by the social cognitive theory of self-regulation.

Mechanism	Number of interventions	Description
**Feedback**	35	Information about behaviour was present from actively using any type of self-monitoring
	26	Information about behaviour was available from instructors who reviewed monitored data, or from data summaries/graphs
**Temporal proximity**	16	Behaviour was monitored in real-time with automatic devices (pedometer, peak air flow, heart rate, blood glucose monitors)
**Consistency**	35	Behaviour was expected to be routinely observed and recorded
**Focus on success**	16	Importance of attending to positive changes was emphasized with positive thinking, recognizing success, and expectations
**Value of behaviour**	28	Importance of the behaviour, its influence on health, and/or importance of self-monitoring the behaviour was emphasized
	5	Option to choose to monitor personally selected behaviour
**Control**	24	Active teaching of skills needed to modify behaviour through personalized problem-solving, development of action or relapse prevention plans, to overcome barriers to change
**Motivation**	24	Identification and setting of goals, behavioural contracts, setting rewards for progress, personal motivational interviewing
	8	Pre-set goals selected by the intervention
**Self-diagnosis**	14	Education about common barriers or facilitators to behaviour
	11	Guided to explore environment and identify personal triggers, barriers or facilitators to behaviour

#### Self-judgement

All of the included interventions incorporated characteristics addressing at least one of the mechanisms related to self-judgement. Each intervention addressed an average of 2.4 of the 5 self-judgement mechanisms. The five self-judgement mechanisms were implemented less comprehensively than those of self-monitoring, with an average of 16.6 interventions addressing each of the five mechanisms. The self-judgement mechanisms ‘Social comparison’ and ‘Statistical comparison’ were infrequently addressed, by 11 and seven interventions, respectively. Table 5 describes the intervention characteristics that addressed each of the self-judgement theory mechanisms. S5 Table provides a summary of how many self-judgement mechanisms were addressed by each of the individual interventions.

**Table 5:** Characteristics of interventions included in the review (n = 35) addressing the self-judgement mechanisms as proposed by the social cognitive theory of self-regulation.

Mechanism	Number of interventions	Description
**Social comparison**	11	Group discussion of progress, problems and solutions, or web-based tracking of selected peers’ progress
**Self-comparison**	20	Encouraged to review progress of monitored behaviour and goals
**Statistical comparison**	7	Compared monitored data with national nutritional or physical activity guidelines to identify differences, or provided with evidence-based data from existing studies
**Modeling**	18	Demonstrations from instructors, scenario examples from materials, or identifying/engaging with a role model
**Education/ reaction**	27	Encouragement, praise, support, and/or feedback on progress from instructors

#### Self-evaluation

We identified a total of 21 interventions that included characteristics related to self-evaluation. Each of these interventions addressed an average of 0.7 of the 3 self-evaluation mechanisms. Self-evaluation was poorly addressed across interventions, with an average of 8.6 interventions addressing each of the three mechanisms. The self-evaluation mechanisms ‘Self-incentives’ and ‘External rewards’ were rarely addressed, by six and four interventions, respectively. Table 6 describes the intervention characteristics that addressed each of the theory mechanisms. S5 Table provides a summary of how many self-evaluation mechanisms were addressed by each of the individual interventions.

**Table 6:** Characteristics of interventions included in the review (n = 21) addressing the self-evaluation mechanisms as proposed by the social cognitive theory of self-regulation.

Mechanism	Number of interventions	Description
**Self-satisfaction**	16	Guided in self-approval or respect for behaviour through promoting confidence, self-efficacy, acceptance, and positive thoughts associated with behaviour change
**Self-incentives**	6	Guided in personally setting self-administered rewards for achieving progress or attaining goals
**External rewards**	4	Rewarded for achieving progress, such as certificates, stickers, t-shirts, bags

### Exploring Differences in Use of the Theory Components

Of the interventions that we evaluated as having a low risk of bias, most (9/13 interventions) used all three of the main theory components. Just over half of the interventions that we evaluated as having high risk of bias (6/11 interventions) and unclear risk of bias (6/11 interventions) used all of the three main theory components.

## Discussion

This review provides an example of how literature can be systematically reviewed to identify the extent to which a selected theory has been used as a framework for existing interventions. To illustrate how researchers can explore theory use for interventions, we provide an overview of the specific theory components and mechanisms that were incorporated into interventions developed using the social cognitive theory of self-regulation as a conceptual framework. From a comprehensive search of multiple sources we identified 202 studies reporting interventions that used the theory, however, only 35 interventions actually incorporated self-monitoring and accurately used the social cognitive theory of self-regulation to develop interventions for the management of arthritis, asthma, diabetes, heart disease, and overweight/obesity. All of the interventions addressed at least two of the main theory components, and 21 of the interventions incorporated characteristics that addressed mechanisms related to all three of the main theory components. We identified that self-monitoring was the theory component used most comprehensively across interventions, with a greater proportion of self-monitoring mechanisms being addressed than those of self-judgement and self-evaluation. Although the self-monitoring mechanisms were often included within interventions, we identified that the self-judgement mechanisms ‘Social comparison’, and ‘Statistical comparison’, and the self-evaluation mechanisms ‘Self-incentives’, and ‘External rewards’ were rarely implemented.

Our review provides a novel example of how to explore the application of theory within existing interventions. Recommendations for the development of theory-driven interventions begin with the suggestion of exploring existing interventions, and conducting a systematic review if relevant synthesized evidence is unavailable for the health condition of interest [5,57]. Reviews exploring theory usually do so by identifying which theories are commonly used [7,8], or by testing theoretical mechanisms associated with change [58,59], rather than identifying intervention characteristics that are used to address theoretical mechanisms. Researchers can use our process as a first step during intervention development to identify which theory mechanisms are commonly or infrequently addressed by interventions, to determine if the selected theory is a feasible framework for development of future interventions for health conditions similar to those included in the review. This type of review is an information source that illustrates examples intervention characteristics used to address theory mechanisms, and can provide direction for use of the characteristics in the development process of future interventions.

Of the 202 full text articles screened, 105 studies evaluating interventions using the social cognitive theory of self-regulation as a conceptual framework were excluded because they did not include self-monitoring as a core component of the intervention and therefore did not appropriately address the self-regulation concept of the theory. Studies evaluating interventions reporting use of the social cognitive theory as a conceptual framework, often either address only specific concepts of the overall theory, or report use of the theory without appropriately including intervention characteristics to approach its theoretical concepts. These findings are similar to those of a review exploring the general use of theory in health behaviour literature, which identified that 70% of all theories were merely mentioned within the research, rather than being appropriately applied [7]. Researchers and clinicians should cautiously interpret individual studies that report using theory as a conceptual framework, as we found many interventions appear to only cite the theory without actually describing how they addressed each of the main theory components. A systematic review as we have conducted can help to highlight the interventions that appropriately implement theory.

In spite of our comprehensive search across multiple sources that identified over 16 thousand citations, we retrieved only 35 self-monitoring interventions developed using the social cognitive theory of self-regulation, with less than ten interventions identified for four of the five health conditions, and only 21 interventions addressing all three of the main theory components. These numbers are not surprising, considering that only 8% of the published health behaviour literature reports interventions that apply theory as a conceptual framework during development [7]. Although our review process was successful for identifying interventions developed using a well-known theory, we did so across five health conditions. Researchers interested in exploring theories that are infrequently implemented, or are exploring uncommon health conditions, may find an insufficient availability of relevant reports evaluating interventions of interest.

The Kappa values reporting our interrater reliability for title and abstract screening, full-text, screening, and risk of bias assessment were moderate, yet lower than preferred. We conducted the title and abstract screening in increasing increments, (100, 200, 500, etc.) with discussion between reviewers at each stage. Although our Kappa values improved with each increment from a starting Kappa of 0.37 from screening 100 abstracts to a final Kappa of 0.71 from screening 1,100 abstracts, our overall Kappa (0.60) is a result of the collective interrater reliability. To improve interrater reliability for title and abstract screening, we recommend that reviewers review small increments of abstracts until a stable and acceptable interrater reliability is reached. When we were screening full-text articles, the reviewers were most often discrepant when determining whether or not self-monitoring was a core component of the intervention. This discrepancy is largely a result of poor reporting within the reviewed publications. As a result of unclear or missing information, the reviewers often independently searched for additional information from available protocols, publications, or online websites that reported information about the intervention. The reviewers sometimes explored different sources of supplemental information, resulting in differing opinions as to whether self-monitoring was a core component of the intervention or not. To avoid this type of discrepancy, we recommend that reviewers decide a priori whether or not they will be searching for additional information. If additional information about an intervention will be searched, reviewers should ensure that the same information is examined by all reviewers involved. When assessing risk of bias, the reviewers consistently identified when an intervention was of high risk. The reviewers were most often discrepant when determining if risk of bias was low or unclear, with one reviewer tending to specify ‘low’, while the other tended to specify ‘unclear’. Although discrepancies were regularly discussed throughout this process, the trend in how the reviewers’ rated (low or unclear) was identified retrospectively. To avoid missing the identification this type of trend, we recommend that reviewers search for any patterns in their ratings while discussing discrepancies in their risk of bias assessment. This type of pattern may also arise when screening title and abstracts or full-texts and could be useful for reviewers to identify early during screening and improve overall reliability by guiding decisions about how to address and prevent further discrepancy.

We attempted to comprehensively retrieve information related to interventions through duplicate publications, available resources, and author contact, however, we were only able to judge intervention characteristics based on available information, often provided as a summary or table of contents. Only five of the authors of included interventions provided us with access to full treatment manuals. We were able to use the additional information to identify more theory mechanisms and components that were not addressed in the published materials that we originally extracted. For example, we identified intervention characteristics guiding participants in self-evaluation in four interventions that were not previously identified as using this theory component. Most publications provided overviews of intervention content with broad overarching concepts. Through supplementing our extraction with information from available manuals, it became clear that publications were not comprehensively representing all of the intervention content and application to theory mechanisms and components that we were able to identify from their manuals. Without the option of reviewing entire intervention manuals for the remaining interventions, it is difficult to confirm that we have comprehensively identified all of the intervention characteristics related to relevant theoretical mechanisms. Poor description of intervention content is recognized as a common problem in the reporting of interventions [60]. To address the problem of underreporting and to improve clarity in the use of theoretically based interventions, intervention investigators should provide access to a comprehensive outline of intervention characteristics and how they apply to each of the related theory mechanisms [61].

Fourteen of the 35 interventions did not address self-evaluation, one of the three main components of the social cognitive theory of self-regulation. We hypothesize that potential reasons for low frequency of identified self-evaluation mechanisms could be that the mechanisms were either more difficult for intervention researchers to implement, or they may have been incorporated but not reported in the available publications. The self-evaluation mechanism ‘External rewards’, addressed by only four interventions, may have been interpreted by developers as too expensive or time consuming to administer and therefore not addressed by the intervention. It is possible that the other two self-evaluation mechanisms were addressed by intervention characteristics that were not explicitly reported in the identified publications. The mechanism ‘Self-satisfaction’ is associated with positively recognizing achievement of progress or goals, and the ‘Self-incentive’ mechanism is associated with setting and administering personal rewards as sources of motivation and reward. For example, when participants were instructed to actively set goals (to address the ‘Motivation’ mechanism), they may also have been guided to react positively to achievement (‘Self-satisfaction’), or set personal rewards to administer upon achievement of the goals (‘Self-incentives’). Therefore the self-evaluation component may have been underrepresented due to availability of resources, or not been identified due to inaccurate reporting. These issues highlight the importance of comprehensive reporting, to improve replicability of similar interventions, and facilitate empirical and clinical understanding of the mechanisms addressed and intervention characteristics used [6]. Future researchers can use protocols and publications about intervention development as a source of understanding the process of which intervention characteristics were selected to address specific theory mechanisms. The development of consensus guidelines for guiding the use of theory within interventions is needed to improve both reporting use of theory use as well as implementation of theory throughout intervention development.

In our subgroup analyses that explored the number of theory components that were addressed according to assessed risk of bias, we found that nine of 11 interventions with low risk of bias incorporated intervention characteristics associated with each of the three main theory components in contrast to only six of 11 and 12 interventions with high or unclear risk of bias, respectively. It is possible that these small differences may have been influenced by poor reporting. Since risk of bias assessment relies on reported information [24], poor reporting may contribute to some interventions being assessed with high or unclear risk of bias and incomplete descriptions of theory mechanisms. These differences highlight the importance of accurate reporting to allow for understanding of mechanisms and intervention characteristics addressed.

### Limitations

This review is not without limitations. We may have overlooked relevant interventions that were developed using the social cognitive theory of self-regulation, but that failed to cite either of the two publications we specified for inclusion. However, we expect that our inclusion criteria identified the best examples of interventions developed using the theoretical framework. We surmise that our database searching, citation searching, systematic review reference list searching, and reference list searching of included studies, limited the number of interventions missed in our investigation.

During our consensus process of determining how we would judge whether or not intervention characteristics address theoretical mechanisms, we identified some overlap in concepts of the theory. As a result of this overlap, we may have been overly inclusive when identifying whether each of the theory components and mechanisms were represented. For example, when participants were instructed to select their own rewards contingent on behavioural progress, the characteristic was judged to apply to both ‘Self-incentives’ of the self-evaluation component, and ‘Motivation’ of the self-monitoring component. The available descriptions of the social cognitive theory of self-regulation theory do not provide guidelines as to which mechanisms may overlap, or outline specifically which mechanisms or combination of mechanisms may be most relevant or useful for successful behaviour change. We therefore attempted to explicitly outline in our coding guide potential overlap across mechanisms, and set our criterion of identifying theory components at a minimum to provide a foundation upon which to build future exploration of applying the social cognitive theory of self-regulation in the development of interventions for chronic health conditions. Research is needed to identify and evaluate which specific mechanisms and associated intervention characteristics are most important to address in behaviour change interventions. These evaluations may lead to the development of comprehensive guidelines suggesting how to use the theory mechanisms and components when developing interventions theory.

### Future Directions

We conducted this systematic review as a first-step method to inform the process that researchers can take during intervention development. Review authors exploring use of the social cognitive theory of self-regulation are encouraged to use our extraction guide to identify intervention characteristics addressing the theory components (S2 Table). For review authors exploring a different theory, following a similar consensus procedure with multiple reviewers to develop an extraction guide that includes comprehensive understanding of the type of intervention characteristics that will be judged as addressing the theory mechanisms may be useful. Our extraction guide may serve as an appropriate starting point and an example of how theory can be identified from exploring intervention characteristics.

If a review identifies that theory is comprehensively addressed across interventions, as our review did for the social cognitive theory of self-regulation for chronic health conditions, sufficient information is likely available for researchers and clinicians to identify which theory mechanisms to consider including during the preliminary phases of developing a theory-driven intervention. Researchers and clinicians can use the review information to choose intervention characteristics that are commonly incorporated to address the theory mechanisms, likely based off of frequency of implementation across interventions. The phases for development of theory-driven interventions suggested by the UK Medical Research Council can then be followed for further testing the intervention components, to identify what intervention version and characteristics can achieve optimum clinical effectiveness [5,6]. If, on the other hand, a review does not identify that theory or specific theoretical mechanisms have been comprehensively used across existing interventions, additional testing and exploration using alternative methods or theories may be required to identify which, if any, of the theory mechanisms are applicable to the population of interest.

We assumed for the purpose of this review that theory-driven interventions provide some benefit over atheoretical interventions. However, the effectiveness of theory-driven interventions compared to those developed without a theoretical framework is unclear. Future research should explore whether there are any benefits when implementing theory-driven in comparison to atheoretical interventions. Even if theory-driven interventions are identified equally as effective as atheoretical interventions, they build on existing knowledge and provide explanations of specific interactions that influence how interventions may work, which is useful for informing improvement and modification of future intervention characteristics and implementation [60].

## Conclusions

The present review establishes that systematic review is a feasible method of identifying use of theory as a conceptual framework for existing interventions. We used the social cognitive theory of self-regulation as an example and identified that it is an adequate and practical theoretical framework to guide the preliminary phases of intervention development for some chronic health conditions. Researchers and clinicians can use this type of systematic review to identify whether a selected theory is a feasible framework to guide intervention development, and which intervention characteristics are used to address the theoretical mechanisms. This work provides a preliminary investigation into exploring use of theory to inform the development of interventions. Further guidelines are needed to assist exploration of theory as a framework in the early phases of intervention development.

## Supporting Information

S1 Table PRISMA Checklist. (DOC)

S2 Table Coding Guide of Intervention Characteristics Addressing the Social Cognitive Theory of Self-regulation. (DOCX)

S3 Table Cochrane Collaboration’s Risk of Bias Tool. (DOCX)

S4 Table Characteristics of Randomized Controlled Trials Evaluating Self-monitoring Interventions for Adults that were Developed Using the Framework of the Social Cognitive Theory of Self-regulation. (DOCX)

S5 Table Frequency of Theory Mechanisms Addressed by at Least One Intervention Characteristic for each Included Intervention. (DOCX)

S1 Text Systematic Review PubMed Search Strategy. (DOCX)
