# Supplementary material for: A Systematic Review Exploring the Social Cognitive Theory of Self-Regulation as a Framework for Chronic Health Condition Interventions
Source: PLoS One. 2015 Aug 7;10(8):e0134977. doi: 10.1371/journal.pone.0134977 (PMC4529200; doi:10.1371/journal.pone.0134977)
Supplement: S2 Table — (DOCX) [file pone.0134977.s002.docx]

**S2 Table. Coding Guide of Intervention Characteristics Addressing the Social Cognitive Theory of Self-regulation**

| **Self-monitoring mechanisms** | | |
| --- | --- | --- |
| **What is the outcome of interest?** | | |
| **Mechanism** | **Theoretical explanation** | **Description of how to recognize theory application in interventions** |
| **Self-monitoring behaviour** | Self-monitoring provides information for the individual to evaluate their ongoing progress, leading to behaviour change. | **Does the intervention explicitly state the behaviour that is being monitored?** |
| **Feedback** | Self-monitoring enhances performance when individuals have clear evidence of progress on the monitored behaviour. Knowledge of how one is doing influences behaviour change, goal-setting, and self-evaluation. | **Does the intervention provide participants with information that allows them to examine their progress on the monitored behaviour?**   - Participants actively self-monitor their behaviour through increased attention and tracking of the behaviour and its patterns - Participants receive information through the use of a diary, data summaries or graphs, or professional guidance |
| **Temporal proximity** | Self-directed change is easier for behaviours that are monitored as they occur, in real-time. Immediate self-monitoring gives continuing information. Attention to ‘distal’ behaviours through recall is less likely to provide useful information or lead to successful behaviour change. | **Does the intervention expect participants to monitor their *behaviour***^1,2^ **close in time to when it happens?**   - Participants expected to monitor behaviour close to when it occurs. - Participants may be requested to monitor behaviour using multiple methods (e.g., monitoring steps taken throughout the day using a pedometer, and recording the total number of steps at the end of the day). When coding temporal proximity, do so using the method that best reflects how the behaviour was measured.   ^1^Participants may be expected to monitor behaviour and outcome. When coding temporal proximity, code monitored behaviour, not outcome  ^2^The type of behaviour that is being monitored is important to consider. If, for example, participants are expected to monitor their daily footsteps - using a pedometer in real-time is an acceptable method of doing so. If, on the other hand, participants are requested to monitor their level of weekly activity - monitoring daily exercise at the end of each day is acceptable proximity to the behaviour. |
| **Consistency** | Regular self-monitoring of behaviour is more informative than intermittent self-monitoring, because it provides continuous attention to performance. | **Does the intervention expect participants to regularly monitor their behaviour?**   - Participants are expected to monitor their behaviour following a specific rule, such as every time it occurs, or on a daily basis (e.g., monitoring every meal, daily activity, exercise) |
| **Focus on success** | Attending to positive behavioural accomplishments is more encouraging for behaviour change than paying attention to failures, which can lower subsequent performance and accomplishments. | **Does the intervention encourage participants to focus on achievements or accomplishments with regard to behaviour change?**   - Participants receive information or training about focusing on positive or successful behaviour changes (e.g., learning to focus on healthy eating choices, instead of focusing on the foods that have been given up) - Participants are guided to implicitly focus on positive changes through rewards for positive progress on their behaviours (rewards direct attention toward achievement) |
| **Value of behaviour** | Behaviours with perceived importance are likely to receive more attention when self-monitored, than are behaviours not perceived to be important. Perceived importance can be established naturally from personal experiences, or it can be taught (externally established), from assigned positive or negative values. | 1. **Does the intervention encourage participants to monitor behaviours that are naturally perceived as important to them?**  - Participants can choose to monitor behaviour that they think is important and worth exploring.  1. **Does the intervention educate participants about the importance of the monitored behaviour (that is predetermined by the intervention) in relation to health outcome?**  - Participants receive instruction on how the behaviour (e.g., physical activity) is important for health outcome (e.g., weight) - Participants receive instruction on the importance of monitoring the behaviour in relation to health outcome |
| **Control** | Self-monitoring is more likely to produce lasting effects in behaviours that are relatively easy for the individual to modify. Self-monitoring only has minor effects on behaviours that are resistant to change. | **Does the intervention actively teach participants the skills necessary to help deliberately modify their behaviour?**   - Participants are taught how to overcome their personal barriers to behaviour change - Active problem-solving is provided about the steps to increase the presence of good behaviours, and decrease unwanted behaviours - Personalized ‘action plans’ or ‘relapse prevention plans’ are created for planning the continuation of behaviour change   Note: Passive education about behaviour change skills is not sufficient to be coded as guiding participants in how to ‘control’ their behaviour |
| **Motivation** | People who want to change the behaviour they are monitoring and who set goals are more likely to react positively to the progress they are making. Similar to ‘value of behaviour,’ motivation can be implicit based on personal experiences, or it can be externally established by instruction about setting goals. | 1. **Does the intervention guide participants in selecting goals related to behaviour change that are personally important to them?**  - Participants are guided in setting their own behaviour change goals - Participants set their own rewards as incentives - Participants develop and sign behavioural contracts  1. **Does the intervention give participants pre-set goals selected by the intervention?**  - Participants are provided with goals for change already set by the intervention (e.g., specific calorie intake) - Participants are made aware of external rewards provided by the intervention that are contingent upon progress |
| **Self-diagnosis** | Self-monitoring behaviour can identify new patterns or insights among situations, thoughts, and actions. Self-insights related to behaviour can lead to behaviour change. | **Does the intervention educate participants about important cues or associations that are linked with or lead to the behaviour or outcome?**   - Participants receive training about the associations between environmental triggers and the onset of a behaviour - Associations, cues, barriers, or facilitators to behaviour are identified (e.g., understanding cues to over-eating) |
| **Self-judgement** | Personal standards guide the appraisal and understanding of the self-monitored information, influencing both self-evaluation and self-directed behaviour change. | **Does the intervention encourage participants to reflect on their behaviour with regard to their own personal standards, in order to judge the progress of the behaviour?**   - Participants identify changes in behaviour (e.g., noticing that physical activity has increased) - Participants identify changes in outcome (e.g., progress with increasing physical activity may lead to a change in weight) |
| **Social**  **comparison** | - Behaviour is easier to regulate when there is some measurement of adequacy/ achievement in comparison to peers, in order to determine a ‘relative’ standing. Social comparison allows for evaluating the progress of behaviour change based on that of peers in similar situations, in particular those with similar capabilities. | **Does the intervention explicitly state that participants are encouraged, or provided with the opportunity to judge the progress of their behaviour change against their peers?**   - Peer group-based discussion of progress is encouraged or facilitated - Competition with peers is encouraged or facilitated - Peer group-based discussion of problems or successes is encouraged or facilitated - Behaviour comparison is encouraged or facilitated through groups |
| **Self-comparison** | Previous behaviour is used as a contrast to ongoing performance. People usually try to surpass their previous accomplishments, seeking self-satisfaction from progressive improvement. | **Does the intervention explicitly encourage or expect participants to judge their *own^1^* progress based on previous behaviour?**   - Participants are encouraged to reflect on their progress - Progress is identified using monitored data - Monitored data is compared with goals - Participants are encouraged to compare previous experiences with ongoing ones (e.g., think back to how you used to feel in comparison to how you feel now)   ^1^Note: Self-comparison is only present if participants are evaluating their behaviour on their own, without any external guidance from an instructor. They are creating their *own* judgements, without any input from others. |
| **Statistical comparison (Normative comparison)** | Evaluating behaviour progress based on normative data using statistics such as percentiles and deviance from the mean can guide the judgement of progress relative to a large sample. However, it is important that the comparison sample is representative of the intervention group. | **Does the intervention provide or guide the comparison of participants’ progress with normative data?**   - E.g., national nutritional intake, or daily activity recommendations - E.g., Evidence-based research providing population-based statistics |
| **Modeling** | Standards can be formed by viewing the performance of others with proven success, and how those others respond to their own behaviour. Forming standards of behaviour progress through examples is especially useful when people have little personal experience in the performance domain. | **Does the intervention provide examples of successful behaviours or encourage the identification of individuals with successful behaviour change, to which participants can compare their progress?**   - Appropriate people are provided as models (e.g., physiotherapist demonstrating the appropriate way to do strength training exercises) - Intervention materials provide examples of models (e.g., DVDs, pictures, vignettes of people) – as long as these are connected to an outcome to guide participants in understanding what is correct/appropriate/successful - Participants are encouraged to identify their own role models |
| **Engagement/ encouragement/ reactions** | Personal standards about the progress of behaviour change can come from the direct guidance/teaching of influential and significant persons. Standards can also be formed based on the reactions of others to the behaviour, especially the reactions of influential and significant persons. | **Does the intervention provide engagement, encouragement, or positive reactions from the individuals leading the interventions, in order to help the participants understand the progress of their behaviour change?**   - Instructors work with participants to identify progress - Instructors inform participants of the progress they have made - Instructors give participants praise for progress or encouragement to continue making improvements |
| **Self-evaluation** | A positive appraisal of oneself can result in a gain in self-respect, leading to behaviour change through reinforcement based on personal standards. | **Does the intervention explicitly encourage and guide participants to personally evaluate their own behaviour?** |
| **Self-satisfaction** | People pursue actions that produce positive self-respect. Anticipated self-approval when personal standards are fulfilled increases the likelihood of performance. | **Does the intervention explicitly encourage or guide participants towards feeling self-approval or self-respect associated with behaviour change?**   - Participants are encouraged to recognize self-efficacy or outcome expectations related to their behaviour - Participants receive education about recognizing goal attainment - Participants are encouraged to change negative thoughts about behaviour to positive thoughts |
| **Self-incentives** | By personally setting tangible rewards that are conditional upon accomplishments, people can get themselves to do things they otherwise would not. | **Does the intervention explicitly encourage participants to personally set and administer their own rewards for behaviour change?**   - Participants are guided to actively set rewards for themselves based on progress |
| **External rewards** | Individuals are more likely to complete behaviours when rewards are contingent on the successful behaviour completion | **Does the intervention set tangible rewards for participants, contingent on behaviour change?**   - Rewards are distributed following behaviour change (e.g., achievement certificates, small gifts) |
