# Supplementary material for: A Systematic Review Exploring the Social Cognitive Theory of Self-Regulation as a Framework for Chronic Health Condition Interventions
Source: PLoS One. 2015 Aug 7;10(8):e0134977. doi: 10.1371/journal.pone.0134977 (PMC4529200; doi:10.1371/journal.pone.0134977)
Supplement: S3 Table — (DOCX) [file pone.0134977.s003.docx]

**S3 Table. Cochrane Collaboration’s Risk of Bias Tool**

| **Domain** | **Support for judgement** | **Review authors’ judgement** |
| --- | --- | --- |
| *Selection bias.* |  |  |
| **Random sequence generation.** | Describe the method used to generate the allocation sequence in sufficient detail to allow an assessment of whether it should produce comparable groups. | Selection bias (biased allocation to interventions) due to inadequate generation of a randomised sequence. |
| **Allocation concealment.** | Describe the method used to conceal the allocation sequence in sufficient detail to determine whether intervention allocations could have been foreseen in advance of, or during, enrolment. | Selection bias (biased allocation to interventions) due to inadequate concealment of allocations prior to assignment. |
| *Performance bias.* |  |  |
| **Blinding of participants and personnel** *Assessments made for each main outcome* | Describe all measures used, if any, to blind study participants and personnel from knowledge of which intervention a participant received. Provide any information relating to whether the intended blinding was effective. | Performance bias due to knowledge of the allocated interventions by participants and personnel during the study. |
| *Detection bias.* |  |  |
| **Blinding of outcome assessment** *Assessments should be made for each main outcome (or class of outcomes)*. | Describe all measures used, if any, to blind outcome assessors from knowledge of which intervention a participant received. Provide any information relating to whether the intended blinding was effective. | Detection bias due to knowledge of the allocated interventions by outcome assessors. |
| *Attrition bias.* |  |  |
| **Incomplete outcome data** *Assessments should be made for each main outcome (or class of outcomes).* | Describe the completeness of outcome data for each main outcome, including attrition and exclusions from the analysis. State whether attrition and exclusions were reported, the numbers in each intervention group (compared with total randomized participants), reasons for attrition/exclusions where reported, and any re-inclusions in analyses performed by the review authors. | Attrition bias due to amount, nature or handling of incomplete outcome data. |
| *Reporting bias.* |  |  |
| **Selective reporting.** | State how the possibility of selective outcome reporting was examined by the review authors, and what was found. | Reporting bias due to selective outcome reporting. |
