# Supplementary material for: A Systematic Review Exploring the Social Cognitive Theory of Self-Regulation as a Framework for Chronic Health Condition Interventions
Source: PLoS One. 2015 Aug 7;10(8):e0134977. doi: 10.1371/journal.pone.0134977 (PMC4529200; doi:10.1371/journal.pone.0134977)
Supplement: S4 Table — (DOCX) [file pone.0134977.s004.docx]

**S4 Table.** **Characteristics of Randomized Controlled Trials^a^ Evaluating Self-monitoring Interventions for Adults^b^ that were Developed Using the Framework of the Social Cognitive Theory of Self-regulation**

| **First Author** | **Description of intervention** | **Outcome** | **Monitored behaviour** | **Use of theory components** | **Risk of Bias** |
| --- | --- | --- | --- | --- | --- |
| **Weight/Obesity** | | | | | |
| Annesi [26] | The Coach Approach: 26-week intervention led by a wellness specialist for severely obese people. Focused on enhancing self-efficacy and self-regulation of exercise adherence through individualized education, exercise sessions, and group-based nutritional dietary control | Controlled eating, weight | -Food and calorie intake | All 3 | Unclear |
| Burke [27] | PREFER: 18-month group-based dietitian and exercise physiotherapist-led behavioural and weight loss intervention to increase self-efficacy and dietary adherence | Weight | -Food intake  -Physical activity | All 3 | High |
| Collins [28] | The Biggest Loser Club: 12-week commercial web-based behaviour change weight loss program targeting behaviour change through self-efficacy, goal setting, and self-monitoring | Weight | -Food intake  -Physical activity | All 3 | Low |
| Gallagher [29] | HEELP (Healthy Eating and Exercise Lifestyle Program): 16-week group-based program with supervised exercise and education sessions on nutrition, exercise, and behaviour change from a professional multidisciplinary team | Weight | -Physical activity | All 3 | High |
| Gray [14] | FFIT (Football Fans In Training): 12-week group-based football club program delivered by community coaching staff. Focused on weight management education, and coach-led physical activity sessions | Weight | -Physical activity | All 3 | Unclear |
| Hollis [30]^a^ | 6-month group-based led by nutritionists and behavioural counsellors to encourage a nutrition-driven eating style, and regular exercise with emphasis on group-based problem solving and social support | Weight | -Food intake  -Physical activity | 2 | Low |
| Kiernan [31] | 20-week group-based weight loss program for women led by intervention staff. Focus on problem-solving and setting goals. Randomized to either 8-week stability module prior to the program or an 8-week problem-solving module after the program | Weight | -Food intake  -Physical activity | All 3 | Low |
| Ma [32] | E-LITE(Elevated Cardiometabolic Risk in Primary Care): 12-week group-based dietitian and fitness instructor-led behaviour change intervention focusing on healthy eating, physical activity, problem solving, and physical activity training | Weight | -Food intake  -Physical activity | All 3 | High |
| Mockus [33] | 20-week dietary self-monitoring intervention for weight loss in children focused on diet modification, physical activity, and behavioural skills | Weight | -Food intake | 2 | High |
| Morgan [34] | SHED-IT: 12-week Internet-based weight loss program for men to make changes in physical activity and dietary behaviours | Weight | -Food intake  -Physical activity | All 3 | Low |
| Morgan [35] | Healthy Dads, Healthy Kids: 12-week group-based program for overweight fathers with children, in order to lose weight and become healthy role models | Weight | -Food intake | 2 | Low |
| Patrick [36] | 1-year web-based weight loss intervention designed to improve diet and physical activity behaviours through goal-setting | Weight | -Physical activity | 2 | Unclear |
| Short [37] | ManUP: 9-month web-based physical activity and nutrition behaviour intervention for men to promote self-monitoring of exercise and diet | Weight | -Food intake  -Physical activity | 2 | High |
| Shuger [38] | LEAN (Lifestyle Education for Activity and Nutrition): 14-week group-based intervention focused on physical activity and nutritional lifestyle changes for weight loss | Weight | -Food intake  -Physical activity | All 3 | High |
| **Diabetes** | | | | | |
| Lawler [39] | Living well with diabetes: 18 month physical activity and dietary behaviour intervention focusing on behavioural skills (self-efficacy, problem-solving, goal setting) for increasing physical activity and reducing energy intake for weight loss in Type II diabetes | Diabetes management | -Food intake  -Physical activity | All 3 | High |
| Liebreich [40] | Diabetes NetPLAY: 12-week web-based and email counseling physical activity behaviour change intervention | Diabetes management | -Physical activity | 2 | Unclear |
| Miller [41] | 10-week nutritional education, food purchasing, and meal planning intervention for older adults with diabetes | Glycaemic control | -Food intake | All 3 | Unclear |
| Nansel [42]^b^ | Diabetes personal trainer: 8-week individualized problem solving intervention for self-management of diabetes in adolescents with type I diabetes | Self-management | -Self-selected | All 3 | Unclear |
| Tan [43] | 12-week educational program focused on enhancing self-efficacy to improve self-monitoring of blood glucose in diabetes management | Glycaemic control | -Blood glucose | 2 | Unclear |
| Tudor-Locke [44] | First Step program: 16-week group-based lifestyle program delivered by physical activity experts to increase activity levels | Health outcomes | -Physical activity | 2 | High |
| Van Dyck [45] | 24-week telephone support psychologist-led behavioural modification program to increase physical activity for sedentary behaviour in Type II diabetes patients | Diabetes management | -Physical activity | All 3 | Low |
| **Heart disease** | | | | | |
| Furber [15] | 6-week pedometer-based, telephone behavioural counselling physical activity intervention focused on increasing self-efficacy, health beliefs, and physical activity goals | Physical activity levels | -Physical activity | All 3 | Low |
| Moore [46] | CHANGE: 12-week nurse-taught group-based lifestyle modification intervention to increase exercise through increasing expectancy, self-efficacy, problem solving, and relapse prevention skills | Long-term exercise | -Physical activity | All 3 | Low |
| Padula [47] | 12-week nurse-led inspiratory muscle training intervention for people with heart failure to increase inspiratory muscle strength | Inspiratory muscle strength | -Inspiratory muscle training intensity, duration, frequency | 2 | Unclear |
| Peterson [48] | 12-month individualized telephone-based intervention focused on enhancing positive affect and self-affirmation to increase physical activity for coronary artery disease | Energy expenditure | -Physical activity | 2 | Low |
| Pinto [49] | 14-week telephone home-based exercise intervention focused on activity counseling and problem-solving about barriers for activity | Activity levels | -Physical activity | 2 | High |
| Shao [50] | 12-week self-management, self-efficacy intervention to improve health outcomes in older people with heart failure | Self-management | -Sodium, fluid intake | 2 | Unclear |
| **Arthritis** | | | | | |
| Hughes [51] | Fit and Strong: 8-week group-based lower extremity osteoarthritis strengthening, fitness walking, and behaviour change intervention | Exercise self-efficacy | -Physical activity | All 3 | Low |
| Kovar [13] | Sidewalkers Walking Program: 8-week group-based hospital fitness walking and behavioural educational program for osteoarthritis of the knee | Functional capacity | -Physical activity | All 3 | Unclear |
| Manning [52] | EXTRA (Education, Self-Management, and Upper Extremity Exercise Training in People with Rheumatoid Arthritis) program: 12-week physiotherapist-led group-based training sessions, with education targeting rheumatoid arthritis knowledge, self-efficacy, and disease self-management, and supervised upper extremity exercises. Participants were asked to complete and monitor daily home-exercise | Arthritis disability | -Physical activity  -Exercise intensity | All 3 | Low |
| Shigaki [53] | RA Help: 10-week web-based online self-management intervention for rheumatoid arthritis to increase self-efficacy and quality of life | Arthritis self-management | -Pain  -Stress | 2 | High |
| **Asthma** | | | | | |
| Baptist [12] | 6-week health educator-led telephone and group-based asthma problem-solving, goal setting intervention | Asthma quality of life and control | -Management barriers | 2 | Low |
| Burkhart [54]^b^ | 16-week individualized nurse-led asthma intervention for children, teaching peak flow meter use and asthma education to improve health outcomes | Asthma control | -Peak airflow  -Asthma  -Medication | All 3 | Low |
| Clark [55] | Women breathe free: 6-month nurse health educator-led telephone counseling asthma intervention for women; focused on the role of sex and gender on asthma, and on problem-solving management skills | Asthma control | -Daily activities | All 3 | Unclear |
| McGhan [56]^b^ | Roaring Adventures with Puff (RAP): 6-week group-based asthma intervention for children to build asthma self-management skills | Asthma control | -Peak airflow  -Asthma symptoms  -Medication | All 3 | High |

^a^Observational study design, ^b^Child or adolescent population
