# Supplementary material for: A Systematic Review Exploring the Social Cognitive Theory of Self-Regulation as a Framework for Chronic Health Condition Interventions
Source: PLoS One. 2015 Aug 7;10(8):e0134977. doi: 10.1371/journal.pone.0134977 (PMC4529200; doi:10.1371/journal.pone.0134977)
Supplement: S5 Table — (DOCX) [file pone.0134977.s005.docx]

**S5 Table. Frequency of Theory Mechanisms Addressed by at Least One Intervention Characteristic for each Included Intervention.**

|  | Self-monitoring mechanisms used (of 8) | Self-judgement mechanisms used (of 5) | Self-evaluation mechanisms used (of 3) |
| --- | --- | --- | --- |
| Average number of mechanisms used | 6.2 | 2.4 | 0.7 |
| **Used all three theory components** | | | |
| Annesi [26] | 6 | 2 | 1 |
| Burke [27] | 7 | 4 | 1 |
| Burkhart [54] | 7 | 2 | 1 |
| Clark [55] | 7 | 1 | 2 |
| Collins [28] | 4 | 3 | 2 |
| Furber [15] | 7 | 1 | 1 |
| Gallagher [29] | 5 | 1 | 1 |
| Gray [14] | 8 | 5 | 1 |
| Hughes [51] | 7 | 2 | 2 |
| Kiernan [31] | 7 | 2 | 1 |
| Kovar [13] | 8 | 4 | 2 |
| Lawler [39] | 8 | 2 | 2 |
| Ma [32] | 7 | 3 | 2 |
| Manning [52] | 7 | 5 | 1 |
| McGhan [56] | 6 | 4 | 1 |
| Miller [41] | 6 | 4 | 1 |
| Moore [46] | 6 | 2 | 1 |
| Morgan [34] | 6 | 4 | 1 |
| Nansel [42] | 7 | 2 | 1 |
| Shuger [38] | 8 | 3 | 1 |
| Van Dyck [45] | 8 | 1 | 1 |
| **Used two theory components** | | | |
| Baptist [12] | 6 | 2 | 0 |
| Hollis [30] | 6 | 2 | 0 |
| Liebreich [40] | 3 | 1 | 0 |
| Mockus [33] | 4 | 1 | 0 |
| Morgan [35] | 5 | 2 | 0 |
| Padula [47] | 5 | 3 | 0 |
| Patrick [36] | 5 | 1 | 0 |
| Peterson [48] | 6 | 2 | 0 |
| Pinto [49] | 7 | 1 | 0 |
| Shao [50] | 5 | 2 | 0 |
| Shigaki [53] | 5 | 1 | 0 |
| Short [37] | 4 | 2 | 0 |
| Tan [43] | 7 | 3 | 0 |
| Tudor-Locke [44] | 7 | 3 | 0 |
