# Supplementary material for: A Systematic Review Exploring the Social Cognitive Theory of Self-Regulation as a Framework for Chronic Health Condition Interventions
Source: PLoS One. 2015 Aug 7;10(8):e0134977. doi: 10.1371/journal.pone.0134977 (PMC4529200; doi:10.1371/journal.pone.0134977)
Supplement: S1 Text — (DOCX) [file pone.0134977.s006.docx]

**S1 Text. Systematic Review PubMed Search Strategy**

((((((((((((((obes*[tw] OR overweight[tw] OR over weight[tw])) OR (heart failure[tw] OR cardiac rehabilitation[tw] OR cardiovascular disease[tw])) OR asthma[MeSH]) OR arthritis[MeSH]) OR Diabetes Mellitus[MeSH]) OR (pain*[tw] OR pain[MeSH])))) AND ((((diary*[tw] OR intervention*[tw] OR therap*[tw])))) AND (self regula*[tw] OR self monitor*[tw] OR self manag*[tw] OR social cognitive theory[tw] OR Bandura[tw] OR behaviour change[tw] OR behavior change[tw]))))))
